# From Defense to Dysfunction: Decoding the Paradox of Emergency Granulopoiesis in Sepsis Pathogenesis

**DOI:** 10.34133/research.1011

**Published:** 2025-12-23

**Authors:** Xiaojing Wu, Shuai Liu, Wenhan Hu, Qingyun Peng, Wei Huang, Jinqiu Ding, Yingzi Huang, Haibo Qiu

**Affiliations:** ^1^ Jiangsu Provincial Key Laboratory of Critical Care Medicine, Department of Critical Care Medicine, Zhongda Hospital, School of Medicine, Southeast University, Nanjing, Jiangsu, China.; ^2^ Department of Emergency Medicine, The First People's Hospital of Lianyungang, Lianyungang, Jiangsu Province, China.

## Abstract

Sepsis is a life-threatening syndrome caused by dysregulated host response that culminates in organ dysfunction. Recognizing that many prior reviews treated neutrophils as largely homogeneous or discussed their functions in isolation, we present a framework centered on emergency granulopoiesis that unifies groundbreaking single-cell multi-omics discoveries, bone marrow niche remodeling, and bedside biomarkers such as the immature-to-total neutrophil ratio and sepsis endotypes to explain the shift from defense to dysfunction. As a hallmark adaptation, emergency granulopoiesis rapidly replenishes neutrophils but paradoxically yields immature neutrophils with impaired chemotaxis, excessive neutrophil extracellular trap formation (NETosis)/reactive oxygen species, and immunosuppressive activity that aggravate tissue damage, organ failure, and late immunoparalysis. We synthesize subset-level diversity and map this diversity to transcriptional shifts coupled with epigenetic and metabolic reprogramming as well as stromal inflammation. Clinically, elevated immature-to-total neutrophil ratios and these immature subsets align with disease severity and mortality, supporting biomarker-guided stratification. Stage-specific therapeutic strategies are evaluated along the continuum of emergency granulopoiesis, calibrating NETosis, and modulating metabolic reprogramming and epigenetics to restore neutrophil homeostasis. Furthermore, we delineate testable priorities for future research such as standardizing immature neutrophil phenotyping across cohorts and defining harmonized immature-to-total neutrophil ratio thresholds. By explicitly centering emergency granulopoiesis and integrating single-cell biology with bedside indicators, this review clarifies why a protective program becomes maladaptive in sepsis and outlines practical avenues for targeted interventions and trial design.

## Introduction

Sepsis, defined as a dysregulated host response leading to life-threatening organ dysfunction, affects approximately 49 million individuals annually with a mortality rate of 30% to 50% [1–5]. Survivors often suffer from long-term physical and cognitive impairments [6]. Despite advancements in antimicrobial therapies and critical care interventions [3–5,7], the underlying pathological mechanisms, particularly immune dysregulation, remain poorly understood.

Neutrophils, comprising 50% to 70% of human circulating leukocytes, serve as the key innate immune defenders through multifaceted mechanisms, including chemotaxis, phagocytosis, degranulation, reactive oxygen species (ROS) production, and neutrophil extracellular trap (NET) formation (NETosis) [8–12]. Under homeostaic conditions, the bone marrow maintains neutrophil homeostasis (about 1 × 10^9^ to 2 × 10^9^ cells/kg/day) via tightly regulated cellular programs and microenvironmental signals [13–17]. Even in early-stage sepsis, the host exhibits neutrophilia, defined as an elevated absolute neutrophil count, and a marked increase in immature neutrophil subsets, which reflects accelerated but dysregulated bone marrow-driven emergency granulopoiesis [18]. Unlike steady-state granulopoiesis, emergency granulopoiesis is marked by hyperproliferation of hematopoietic stem/progenitor cells (HSPCs), abbreviated maturation, aberrant expression of cellular markers (e.g., reduced CD10/CD16 expression), and atypical nuclear morphology (e.g., banded nuclei) [19,20]. This accelerated progenitor differentiation and altered developmental pathways facilitate the rapid mobilization of a phenotypically heterogeneous cell population [21]. Elevated levels of immature neutrophils are independently associated with increased disease severity and poor clinical outcomes in patients with sepsis [8,18,22,23].

Nevertheless, emergency granulopoiesis in sepsis is a double-edged sword: Replenishing neutrophils enhances pathogen clearance; conversely, immature neutrophils with defective chemotaxis, dysregulated NETs/ROS production, and immunosuppressive subsets exacerbate tissue damage and may contribute to multiple organ dysfunction syndrome (MODS) [24–26]. This functional paradox highlights the complexity of granulopoiesis and ongoing clinical debates [27]. Most existing reviews catalog infection-driven neutrophil functional changes, including the NETosis and ROS generation, phagocytic capacity, and T cell immunosuppression, often considering neutrophils as largely homogeneous without integrating their heterogeneity with clinical anchors [19,27,28]. To address this gap, this review centers on emergency granulopoiesis, integrating single-cell evidence, bone marrow niche remodeling, and bedside biomarkers, such as the immature-to-total neutrophil (I/T) ratio and sepsis endotypes, to explain how neutrophil responses shift from defense to dysfunction across time and tissues during sepsis. We then evaluate stage-specific therapeutic strategies that target this maladaptive program and align with biomarker-guided stratification. The overall goal is to deepen the mechanistic understanding of sepsis pathophysiology and provide translational guidance for stratified trial design.

## Concept and Research History of Emergency Granulopoiesis

### Steady-state granulopoiesis

**Fig. 1.**
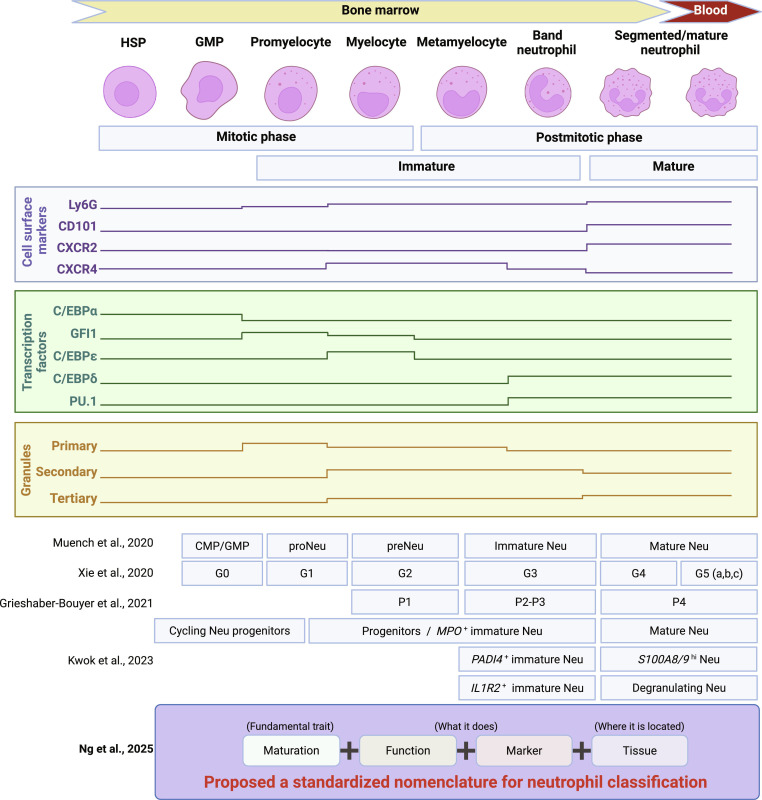
Steady-state granulopoiesis and cross-mapping of neutrophil states. Granulopoiesis is a stepwise program spanning precursor identity, proliferative capacity, master transcription factors, nuclear morphology, staged granule biogenesis, surface proteome, and effector functions, and finally distributed to diversity of tissues. Building on single-cell frameworks, the panel aligns classical maturation stages with different cell surface markers, transcription factors, granules, and commonly used phenotypic/functional markers to harmonize nomenclatures across studies. Note that granule panels distinguish transcript dynamics from protein/organellar stores; secondary and tertiary granules are retained in segmented neutrophils and can be mobilized upon activation. Markers along maturation are shown as gradients rather than absolute levels; band cells and segmented neutrophils are explicitly annotated to align with main-text terminology. GMP, granulocyte–monocyte progenitor; proNeu, proneutrophil; preNeu, preneutrophil; PU.1, purine-rich box-1; C/EBPα/ε/β, CCAAT/enhancer-binding protein α/ε/β; GFI1, growth factor-independent 1; CXCR2/CXCR4, C-X-C motif chemokine receptors 2/4; CD, cluster of differentiation (e.g., CD117, CD81, CD11b, CD101, and CD62L); Ly6G, lymphocyte antigen 6 complex locus G. Figure created with BioRender.com

Under homeostasis, neutrophils arise from hematopoietic stem and progenitor cells through a stepwise maturation cascade: multipotent progenitors (MPPs)→granulocyte–monocyte progenitors (GMPs)→myeloblasts→promyelocytes→myelocytes→metamyelocytes→band cells (immature)→segmented neutrophils (mature) [5,29–31]. This progression is governed by core transcription regulators, beginning with C/EBPα, transitioning to GFI1 and C/EBPε during early myeloid differentiation, and culminating in the activation of PU.1 and C/EBPδ in late-stage granulopoiesis [13,19,27,28,32], together with time-dependent waves of granule biogenesis, from primary (azurophilic) to secondary (specific) and tertiary (gelatinase) [33]. These conventions are consistent with the expression of surface markers, including the increase of CD11b during maturation and egress competence, Ly6G tracking late granulopoiesis, and CD10/CD16 increasing with nuclear segmentation and functional fitness [34]. Moreover, the steady-state granulopoiesis places the CXCR2/CXCR4 axis at the core trigger of release versus retention [35], establishing a clear baseline for comparison with the accelerated programs observed in sepsis-induced emergency granulopoiesis (Fig. 1).

### Research history and milestone discoveries

Research on granulopoiesis has evolved breakthroughs over the past century. Across the early 20th century, foundational works collectively established that the neutrophil in sepsis exhibited a “left shift” immature nuclear morphology based on nuclear lobulation and served as an initial diagnostic marker for infection severity and prognosis [36,37]. From the 1980s to 2010s, studies established that infectious cues [e.g., lipopolysaccharide (LPS) and interleukin-1 (IL-1)] stimulate bone marrow stromal cells to produce granulocyte colony-stimulating factor (G-CSF)/granulocyte–macrophage colony-stimulating factor (GM-CSF) and promote neutrophil mobilization via CXCL12-CXCR4 remodeling, defining a stromal–cytokine axis in host defense [15–17,38]. In 2006, C/EBPβ was identified as the core transcriptional regulator of this emergency output, redefining “bone marrow suppression” as “accelerated compensatory hematopoiesis” [39]. This work was foundational to Manz and Boettcher’s [18] formal coining of the term “emergency granulopoiesis” in 2014, capturing its dual roles in immunity and pathology. Recent single-cell studies clarify neutrophil heterogeneity across homeostasis and sepsis. In mouse models, Xie et al. [33] profiled resting neutrophils in homeostasis and *Escherichia coli* peritonitis conditions and partitioned them into bone marrow stages G0 to G4 and peripheral G5a to G5c. Grieshaber-Bouyer et al. [40] defined the “neutrotime” transcriptional signature, referenced as P1 to P4 along this continuum. Integrative work using single-cell RNA sequencing (scRNA-seq) with cellular indexing of transcriptomes and epitopes by sequencing (CITE-seq) and assay for transposase accessible chromatin with high-throughput sequencing (scATAC-seq) to map stages spanning proNeu, preNeu, immature, and mature neutrophils in detail [41]. In patients, Kwok et al. [19,22] built a whole-blood single-cell multi-omics atlas that identifies immunosuppressive mature and immature neutrophil populations (e.g., IL1R2^+^, PADI4^+^, and MPO^+^) linked to emergency granulopoiesis. To harmonize terminology, Ng et al. [42] proposed a 4-tier framework: developmental stage, functional module, identifier marker(s), and anatomical distribution, with an emphasis on functional validation for assignments. Complementing this, zebrafish studies in 2023 introduced neutrophil “timer” reporters, enabling ratiometric, high-throughput visualization of emergency granulopoiesis dynamics during infection [43].

**Fig. 2.**
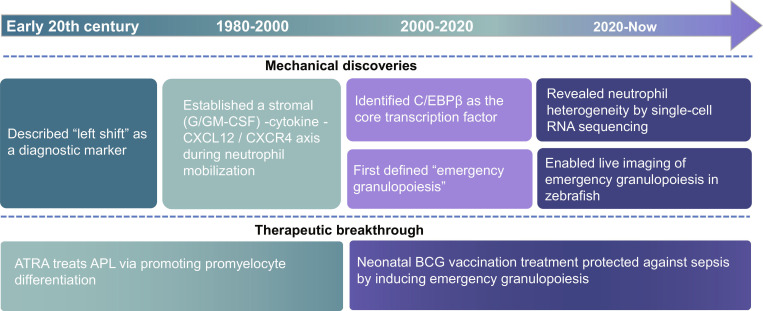
Research history and milestone discoveries of emergency granulopoiesis. The timeline integrates research milestones: from early observations of “left-shift” in hematopoiesis, through the delineation of stromal–cytokine regulatory networks (e.g., G/GM-CSF) and trafficking axes (e.g., CXCL12/CXCR4) (1980s to 2010s), to the development of ATRA differentiation therapy (1988), the identification of the transcription factor C/EBPβ (2006), and the formalization of emergency granulopoiesis (EG) (2014)—this timeline illustrates how cues derived from infection and stress remodel the production and mobilization of neutrophils. Building on this foundation, advances in the 2020s—including the construction of single-cell atlases, innovations in live imaging, and insights into neonatal BCG-associated protection—have deepened our understanding of the underlying mechanisms.

Therapeutically, neonatal Bacille Calmette–Guérin (BCG) vaccination has been shown to confer early protection against sepsis by rapidly inducing emergency granulopoiesis and expanding the neutrophil pool [44,45]. In a landmark 1988 study of Wang and colleagues [46], all-trans retinoic acid (ATRA) has driven terminal differentiation of leukemic promyelocytes, sharply reducing early deaths of acute promyelocytic leukemia (APL). Together, these milestones bridge foundational mechanisms to 3 core translational practices, including the establishment of single-cell maps, the application of live imaging, and the development of emerging therapies for infection-driven emergency granulopoiesis (Fig. 2).

### Definition and biological characteristics of emergency granulopoiesis

**Fig. 3.**
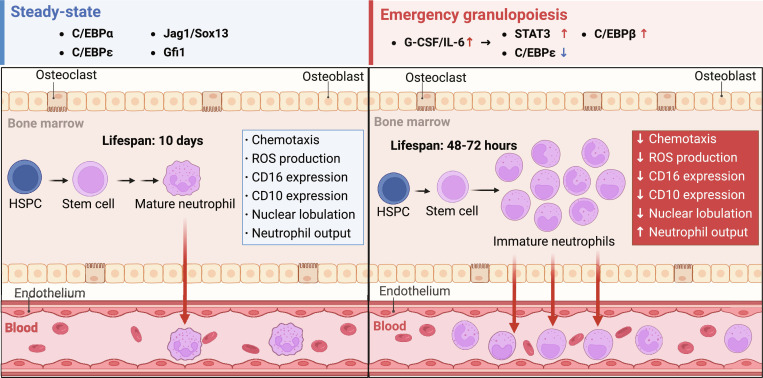
Steady-state versus emergency granulopoiesis under systemic inflammation. Schematic comparison of baseline hematopoiesis with systemic inflammation-triggered emergency granulopoiesis, in which emergency granulopoiesis accelerates neutrophil output, shortens maturation, and promotes premature egress of immature/low-density states. These shifts coincide with coordinated changes in core transcription factors, trafficking receptors, lifespan, nuclear segmentation, surface marker profiles, and effector readouts, consistent with single-cell-resolved maturation continua and inflammation-biased mobilization. ROS, reactive oxygen species; G-CSF, granulocyte colony-stimulating factor; IL-6, interleukin-6. Figure created with BioRender.com

Emergency granulopoiesis is a rapid myelopoietic response to infection, trauma, or inflammatory stress, increasing neutrophil output several-fold above steady-state levels [18,27,47]. Key characteristics of emergency granulopoiesis include the following: (a) accelerated proliferation and differentiation of HSPCs and GMPs, reducing granulocyte cycle from 10 d (steady-state) to 48 to 72 h [19,20]; (b) partial bypassing of maturation checkpoints, leading to premature release of immature granulocytes into peripheral blood [21,48]; (c) production of granulocytes exhibits heterogeneous phenotypes encompassing nuclear morphology (e.g., variable lobulation) and molecular expression (e.g., surface markers), including myelocytes, metamyelocytes, and band cells, alongside diminished CD10/CD16 expression and compromised chemotaxis or ROS generation [25,33]; (d) at the molecular level, emergency granulopoiesis entails reprogramming of hematopoietic regulatory networks. Unlike *Cebpa*-governed early stage of steady-state granulopoiesis, transcription factors *Cebpb* and *Stat3* orchestrate emergency granulopoiesis via G-CSF and IL-6 cytokine cascades [39,49,50]. This transcriptional rewiring prioritizes rapid granulocyte output over functional precision, establishing a “quantity-over-quality” compromise that balances pathogen clearance and immunopathology [38,51] (Fig. 3).

## Impact of Emergency Granulopoiesis on Neutrophil Phenotype and Function

Sepsis-induced emergency granulopoiesis not only compensates for the depletion of mature neutrophils but also dramatically reprograms neutrophil biology [18]. The resulting immature neutrophils display distinct phenotypic, functional, and immunoregulatory characteristics compared to their mature counterparts, forming the cellular basis for sepsis-induced immune dysregulation.

### Emergency granulopoiesis alters neutrophil phenotype and developmental trajectory

Immature neutrophils in septic patients exhibit classical left-shift morphology, with banded or reniform nuclei accounting for up to 45% of circulating neutrophils (normal <5%). These cells contain increased azurophilic granules (occupying 67% of cytoplasmic volume versus 32% in mature cells) and exhibit significantly reduced chromatin condensation, as measured by contour ratio analysis. Surface marker profiling of these immature neutrophils reveals an absence of CD10 and markedly reduced CD16 (FcγRIIIb) expression. Liu et al. [52] identified CD10 as a sepsis-specific marker. scRNA-seq has further revealed the molecular heterogeneity of immature neutrophils, with Hong et al. [53] identifying at least 3 pathogenic subsets: immature inflammatory S100A8/A9^high^ blood, mature metabolic ARG1^high^ blood, and immature immunosuppressive PD-L1^high^ subtypes. These subsets additionally exhibit metabolic rewiring, such as up-regulated glycolytic enzymes HK2 and pyruvate kinase M2 (PKM2), which align with their distinct functional profiles in inflammation and immunosuppression. More importantly, sepsis induces profound reprogramming of granulopoiesis: Sepsis shortens postmitotic maturation from 24 h to 12 to 16 h via accelerated cell cycle progression and simplified differentiation checkpoints [19,20]. Unlike linear maturation in steady-state hematopoiesis, sepsis promotes nonlinear neutrophil development with branching trajectories. Pseudotime mapping shows 35% of bone marrow progenitors deviating into immature proliferating CD74^+^S100A8/A9^high^ bone marrow emergency subsets with enhanced IGF1R/mTORC1 signaling and preserved CXCR2-dependent chemotaxis [19,28]. Therefore, sepsis results in the coexistence of a broader spectrum of developmental stages in both bone marrow and peripheral blood, ranging from promyelocytes to mature neutrophils. This “developmental asynchrony” reflects the relaxation of quality control mechanisms under emergency conditions [27,52].

### Pathophysiological impact of immature neutrophils generated during emergency granulopoiesis

Immature neutrophils in sepsis function as pathological effectors rather than developmentally arrested cells [19,27]. These sepsis-derived immature neutrophils display impaired migration, correlating with the pathogenesis of sepsis. Our recent study demonstrates that sepsis triggers substantial expansion of an immature neutrophil subpopulation exhibiting reverse transendothelial migration (rTEM) capability. This subpopulation disrupts systemic endothelial barrier integrity, thereby mediating sepsis-induced multiorgan injury [54]. On the other hand, Sônego et al. [29] characterized “neutrophil paralysis” as elevated circulatory counts with diminished infection site trafficking. In sepsis-associated acute lung injury (ALI), immature neutrophils accumulate in pulmonary microvasculature through Mac-1-dependent retention, inducing microcirculatory compromise and hypoxemia [29,55]. Functionally, immature neutrophils demonstrate 40% reduced phagocytic efficiency against bacterial pathogen such as *Staphylococcus aureus* and *E. coli* versus mature counterparts [56]. Although displaying elevated basal ROS, these immature neutrophils coexist with impaired stimulus-induced burst capacity [56,57]. This pathological state is associated with NADPH (reduced form of nicotinamide adenine dinucleotide phosphate) oxidase subunit dysregulation (gp91phox suppression) and antioxidant enzyme induction [superoxide dismutase 2 (SOD2) elevation], exacerbating tissue oxidative injury while compromising pathogen clearance [58–60]. In addition, pathogenic NETosis is amplified through peptidylarginine deiminase 4 (PAD4)-dependent histone citrullination following platelet activation, directly propagating sepsis-associated coagulopathy [61]. Inhibition of granulopoiesis by myeloid-specific signal transducer and activator of transcription 3 (STAT3) knockout or G-CSF-neutralizing antibodies alleviates liver and kidney injury and reduces neutrophil infiltration in the lung [52].

### Immunosuppressive mechanisms mediated by sepsis-induced immature neutrophils

In addition to their intrinsic functional abnormalities, immature neutrophils orchestrate immunosuppression via interlinked signaling pathways, thereby promoting the progression of late-stage immunoparalysis in sepsis. Specifically, these cells potently inhibit CD4^+^/CD8^+^ T cell proliferation and effector functions via multiple mechanisms [62]. Qi et al. [23] demonstrated that adoptively transferred CD11b^+^Ly6G^+^CD101^−^ immature neutrophils from septic mice induce T cell anergy and multi-organ failure in recipients, an effect absent in mature neutrophils. Immunosuppressive immature neutrophils, highly expressed PD-L1, IDO1, CD274, and CD86, suppress T cell responses through secretion of inhibitory cytokines [IL-10 and transforming growth factor-β (TGF-β)] and ROS [20,63]. It is worth noting that immature neutrophils in sepsis patients persistently secrete proinflammatory cytokines [IL-8 and tumor necrosis factor-α (TNF-α)] and damage-associated molecular patterns (DAMPs) (S100A8/A9 and HMGB1), which may partially contribute to sepsis-induced immune paralysis and prolonged pathological tissue destruction [56,64]. Immature neutrophil expansion also correlates with the expansion of myeloid-derived suppressor cell (MDSC) [65]. Coudereau et al. [66] identified phenotypic and functional convergence between CD10^low^CD16^low^ immature neutrophils and granulocytic MDSCs (G-MDSCs), characterized by arginase-1-mediated immunosuppression. Collectively, sepsis-induced granulopoiesis emerges as a pathological amplifier rather than compensatory adaptation [19,66] (Table 1). In this review, immature neutrophils are defined phenotypically (human CD10^−^CD16^low^, mouse CD11b^+^Ly6G^+^CD101^−^), whereas G-MDSCs require both an immature/low-density phenotype and demonstrated functional immunosuppression [e.g., T cell suppression, ± ARG1/PD-L1/ROS/nitric oxide (NO) evidence].

**Table. 1.** Differences between mature and immature neutrophils in sepsis

Characteristics	Mature neutrophils	Immature neutrophils	Refs.
Morphological characteristics	Normal nuclear lobulation, banded/reniform nuclei <5%	Marked left shift, banded/reniform nuclei up to 45%	[56]
	Azurophilic granules occupy 32% of cytoplasmic volume	Azurophilic granules occupy 67% of cytoplasmic volume	[19]
	High chromatin condensation (high contour ratio)	Reduced chromatin condensation (low contour ratio)	[19]
Surface markers	CD10^+^, CD16^high^	CD10^-^, CD16^low^	[52]
	Ly6G^high^, CD11b^+^, CD101^+^	Ly6G^low^, CD11b^low^, CD101^−^	[23]
Developmental trajectory	Linear differentiation	Nonlinear branching differentiation, emergence of emergency subsets like CD74^+^S100A8/A9^high^	[28]
	Maturation time 24 h	Maturation time shortened to 12–16 h	[20]
	Single developmental stage (predominantly mature cells)	Diverse developmental stages coexist (from promyelocytes to mature cells)	[19,27]
Functional features	Normal migration, effective chemotaxis to infection sites	Impaired migration (“neutrophil paralysis”, high circulatory counts but reduced infection site accumulation)	[29,54,55]
	High phagocytic efficiency (40% higher than immature cells for bacterial phagocytosis)	Significantly reduced phagocytic efficiency	[56]
	Normal ROS response (strong stimulus-induced burst capacity)	Elevated basal ROS with defective stimulus-induced burst capacity	[56,57]
	Moderate NETosis, no induction of coagulopathy	PAD4-dependent enhanced NETosis inducing coagulopathy	[61]
	Less cytokine/chemokine secretion	Sustained secretion of proinflammatory cytokines (IL-8, TNF-α) and DAMPs (S100A8/A9, HMGB1)	[56,64]
Immunoregulatory mechanisms	No immunosuppressive function	Suppress CD4^+^/CD8^+^ T cell proliferation via PD-L1/IDO1/CD274	[20,23,63]
		Induce T cell anergy via secreting IL-10, TGF-β, and ROS	[23,62]
		Ectopic expression of MHC class II and costimulatory molecules (CD80/CD86) triggering inefficient antigen presentation	[72,74]
		Phenotypic and functional convergence with granulocytic MDSCs (G-MDSCs), mediating immunosuppression via arginase-1	[65,66]

## Systemic Immune Dysregulation Driven by Sepsis-Induced Emergency Granulopoiesis

Sepsis-induced emergency granulopoiesis enhances myeloid responses to infection while systemically suppressing other immune lineages, a process mediated by hematopoietic niche remodeling and epigenetic reprogramming [18,19]. This compensatory mechanism prioritizes innate immunity at the expense of adaptive immune function, reallocating resources to maintain hematopoietic homeostasis under pathological stress.

### Myeloid lineage expansion and lymphopoiesis suppression

Persistent inflammatory signaling in sepsis reprograms HSPC differentiation toward myeloid lineages. The IL-6/Janus kinase (JAK)/STAT3 pathway up-regulates GM-CSF receptor (GM-CSFR) on HSPCs while suppressing *Flt3* transcription, inhibiting lymphoid progenitor expansion [39,67]. Consistent with a progenitor-level bias, Kwok et al. [68] identified that early committed neutrophil progenitors (CMPs) and GMPs expand specifically during the early septic phase, thereby skewing myeloid differentiation toward neutrophils with a concomitant reduction in monocytic output. Conversely, the C/EBPβ–PU.1 complex occupies 80.5% of HSPC chromatin and competitively blocks IKAROS-mediated lymphoid gene activation [49,69,70].

### Monocyte-macrophage exhaustion and polarization defects

Emergency granulopoiesis also disrupts monocyte production through resource competition [18,39]. Monocyte–dendritic cell progenitors (MDPs) experience a transient expansion within 24 h to satisfy the demand for tissue macrophage replenishment [71]. However, prolonged activation depletes the MDP reserves, resulting in a 55% reduction in monocyte output and dramatically down-regulating human leukocyte antigen (HLA)-DR expression, thereby impairing antigen presentation capacity of macrophages [72–74].

### Erythropoietic suppression and iron dysregulation

**Fig. 4.**
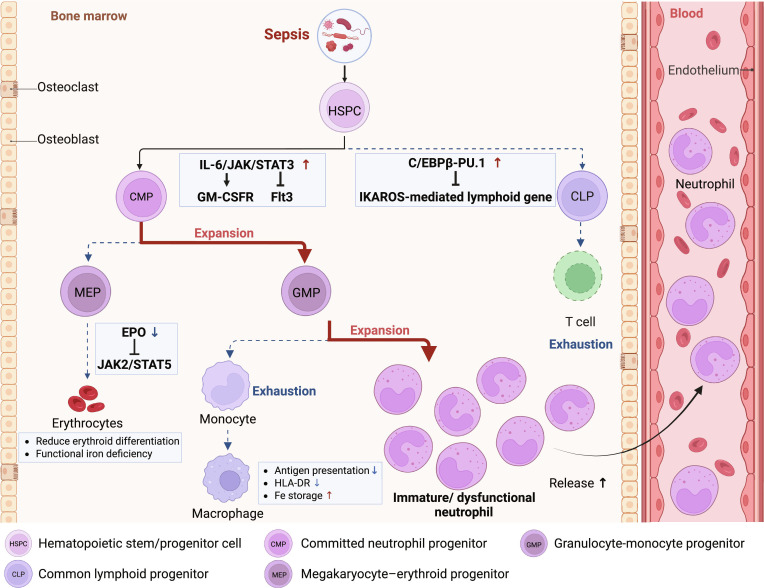
Systemic immune dysregulation driven by sepsis-induced emergency granulopoiesis. Integration across bone marrow–blood–tissue compartments shows how sepsis-related inflammatory cues drive hematopoiesis toward myeloid expansion, accelerate neutrophil production, premature egress, and reshape maturation state, lifespan, nuclear segmentation, CD10/CD16 profiles, and effector outputs. In parallel, emergency granulopoiesis-coupled signaling suppresses lymphopoiesis and disrupts erythropoiesis as well as iron homeostasis. GMP, granulocyte-monocyte progenitor; CLP, common lymphoid progenitor; G-CSF/GM-CSF, granulocyte/granulocyte-macrophage colony-stimulating factor; IL-6, interleukin-6; EPO, erythropoietin. Figure created with BioRender.com.

Emergency granulopoiesis suppresses erythropoiesis via unliganded erythropoietin (EPO) receptor activation of JAK2/STAT5, raising erythroid differentiation thresholds. Concurrently, inflammation lowers serum iron levels and increases ferritin storage in macrophage, inducing functional iron deficiency and reducing red blood cell production in the bone marrow [18–20,39,75,76] (Fig. 4).

## Regulatory Mechanisms of Emergency Granulopoiesis in Sepsis

### Spatiotemporal remodeling of the bone marrow microenvironment

Under septic conditions, the bone marrow undergoes significant microenvironmental reorganization, disrupting hematopoietic niche homeostasis [77]. Within 6 to 24 h post-infection, circulating pathogen-associated molecular patterns (PAMPs) and DAMPs infiltrate the bone marrow cavity through paravascular diffusion or direct vascular leakage [24]. The bone marrow mesenchymal stem cells (MSCs) transit from their steady-state CXCL12^+^ phenotype to CXCL12-abundant reticular (CAR) cells and adopt an IL-6^+^/G-CSF^+^ proinflammatory phenotype upon LPS stimulation. Moreover, single-cell spatial transcriptomic analyses reveal that the CAR cells in the bone marrow reduce CXCL12 secretion by 80% and increase IL-6 and GM-CSF production 5- to 8-fold [34,40]. This phenotypic shift directly promotes myeloid differentiation of HSPCs [78].

Additionally, vascular remodeling critically facilitates microenvironmental adaptation. In vivo 2-photon microscopy shows bone marrow sinusoidal dilation within 12 h post-infection in septic mice, accompanied by a 45% reduction in endothelial tight junction protein Claudin-5 expression [33,79]. The dilated sinusoids and diminished Claudin-5 enhance vascular permeability, accelerating the egress of immature granulocyte. Furthermore, the up-regulation of E-selectin on activated endothelial cells interacts with α4β1 integrins on CD82^+^ immature neutrophils, establishing a specialized rolling–arrest–extravasation axis that mobilizes promyelocytes and myelocytes to peripheral blood within 24 h of sepsis [21,68,80].

**Fig. 5.**
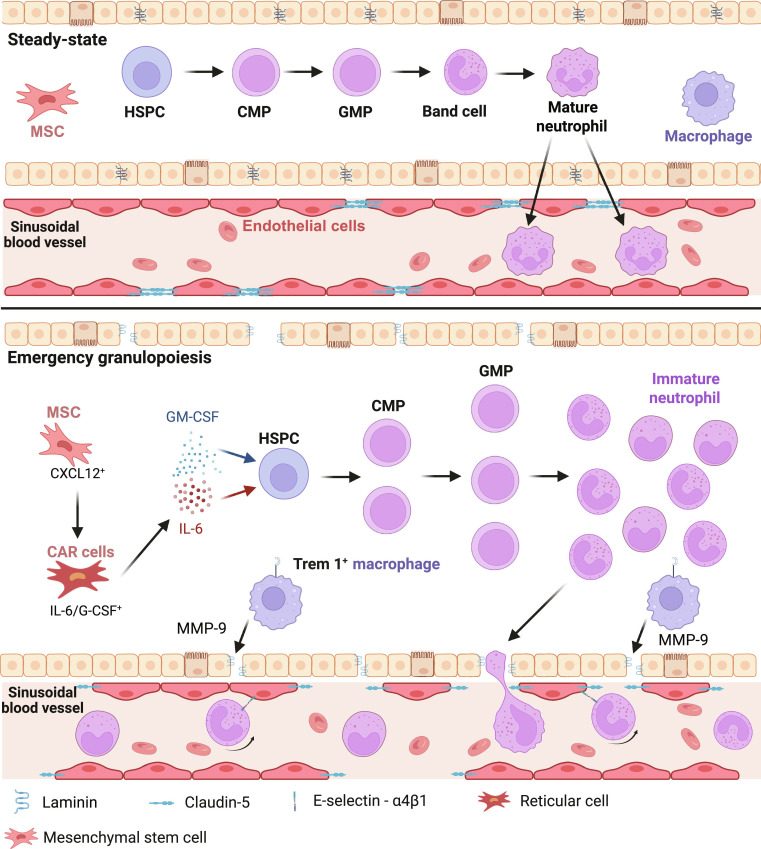
Spatiotemporal remodeling of the bone marrow niche and neutrophil egress in sepsis. Integration of endothelial, stromal/mesenchymal, and perivascular elements shows how systemic inflammation signals reshape the marrow microenvironment during sepsis. CXCL12-producing CAR/MSC populations reduce CXCL12 while acquiring an IL-6/G-CSF-secreting phenotype; vascular leakage and paravascular diffusion increase niche exposure to inflammatory cues; and altered sympathetic/chemokine tone shifts HSPC and GMP fate toward myelopoiesis. These changes collectively accelerate granulopoiesis, shorten maturation, and promote premature egress of immature neutrophil states. MSC, mesenchymal stromal cell; MMP-9, matrix metalloproteinase-9. Figure created with BioRender.com.

Stromal–immune crosstalk in the bone marrow exemplifies a “niche deconstruction–reconstruction” mechanism that provides biophysical and biochemical support for emergency granulopoiesis [18,27]. Macrophages residing in the bone marrow detect inflammatory signals through TREM-1 receptors and secrete matrix metalloproteinase-9 (MMP-9), which degrades laminin and reduces the spatial confinement of hematopoietic progenitors [19,81]. Simultaneously, osteoclast activity increases within 48 h post-infection, driven by amplified coordinated Ca^2+^/RANKL signaling that promotes myeloid differentiation [20,39,82] (Fig. 5 and Table 2).

**Table. 2.** Regulatory mechanisms of granulopoiesis in steady-state versus sepsis

Kind of regulation	Steady-state granulopoiesis	Emergency granulopoiesis in sepsis	Refs.
Cytokine profile	Maintained by low-level G-CSF for homeostasis	Marked by explosive increases in G-CSF, GM-CSF, and IL-6, inducing a cytokine storm	[12,31,33]
Niche microenvironment	Stromal cells secrete homeostatic signals (SCF, CXCL12)	Activated stromal cells secrete pro-inflammatory factors (IL-6, G-CSF) with increased vascular permeability (sinus dilation, Claudin-5 down-regulation)	[15,17,68]
Transcriptional regulation	Dominated by C/EBPα with PU.1 co-activation	Driven by C/EBPβ and STAT3 via inflammatory signaling, prioritizing progenitor proliferation over functional maturation	[13,31,32]
Epigenetic regulation	Stable methylation patterns and lineage-specific gene expression	Dynamic demethylation/acetylation, increased chromatin accessibility, and activation of emergency response genes	[13,35,75]
Metabolic signature	Predominantly oxidative phosphorylation	Enhanced glycolysis with aerobic glycolysis (Warburg effect)	[12,31,47]
Developmental kinetics	Maturation over 5–7 d with strict differentiation checkpoints	Shortened maturation to 2–3 d, bypassing some checkpoints and releasing immature granulocytes (e.g., band cells)	[13,14,29]

### Integrated immune response of hematopoietic stem cells to pathogen stimulation

During sepsis, HSPCs exhibit pathogen-specific transcriptional and functional reprogramming, integrating microbial signals to modulate hematopoietic output. They differentially express pattern recognition receptors (PRRs) in response to different stimulation: GMPs up-regulate Toll-like receptor 4 (TLR4), which activates the MyD88/TRIF pathways, leading to an 8-fold increase in C/EBPβ expression within 30 min [19,20,39]. Meanwhile, inhibiting STAT3/C/EBPβ or TLR4–MyD88–nuclear factor κB (NF-κB) pathways enhance granulocyte maturity, alleviates liver and kidney injury, and reduces neutrophil infiltration in the lung, suggesting that transient suppression of overactivated signals improves outcomes [38,49]. In contrast, viral RNA triggers the RIG-I–MAVS axis, activating IRF7 to divert HSPCs toward plasmacytoid dendritic cell differentiation [83]. This dichotomy highlights the distinct roles of TLR4-driven granulopoiesis in bacterial infections and RIG-I-mediated suppression in viral contexts [18].

Another key feature of emergency granulopoiesis is the hierarchical transcriptional and epigenetic networks of HSPCs. Unlike steady-state differentiation governed by the C/EBPα–C/EBPϵ–PU.1 axis, acute response establishes a distinct STAT3–C/EBPβ regulatory core [20,39]. It is worth noting that the expression of all the members of the C/EBP family was markedly down-regulated in response to infection, with the exception of up-regulated C/EBPβ. Both *Cebpb* and *Stat3* knockout mice exhibited impaired granulopoiesis after G-CSF/GM-CSF and IL-3 stimulation. Chromatin immunoprecipitation sequencing (ChIP-seq) reveals C/EBPβ binding at 1,352 loci in GMPs within 2 h post-infection, 78% proximal to myeloid genes (e.g., CSF3R) [39,69]. Meanwhile, STAT3 directly binds to the promoter region of mice *Cebpb* through a nonclassical binding site. *Stat3* knockout mice were unable to induce expression of C/EBPβ as well as C/EBPβ-dependent granulopoiesis. Moreover, sustained STAT3 phosphorylation (12-fold increase) activates BCL2L1 and CCND3 via glucocorticoid receptor (GR) complex assembly, promoting survival and proliferation [19,20,84]. Epigenetic dynamics further refine transcriptional effects. MLL1 recruitment triples H3K4me1 at granulocyte enhancers, while DNA methylation profiling reveals 2,145 differentially methylated regions in CD34^+^ cells, including hypomethylated DEFA1B linked to immature granulocyte release [19,23,26,51,72].

Noncoding RNA networks also add regulatory complexity. The long noncoding RNA Lnc-EG1 exhibits a 17-fold increase in septic granulocyte precursors and stabilizes Cebpb mRNA through RNA–DNA triplexes, extending its half-life to 8.5 h. Moreover, the circular RNA circHipk3 sequesters miR-124-3p, which leads to the derepression of KLF5, thereby activating terminal differentiation genes such as LCN2. These integrated layers synchronize granulopoiesis with infection dynamics [19,51,84,85].

**Fig. 6.**
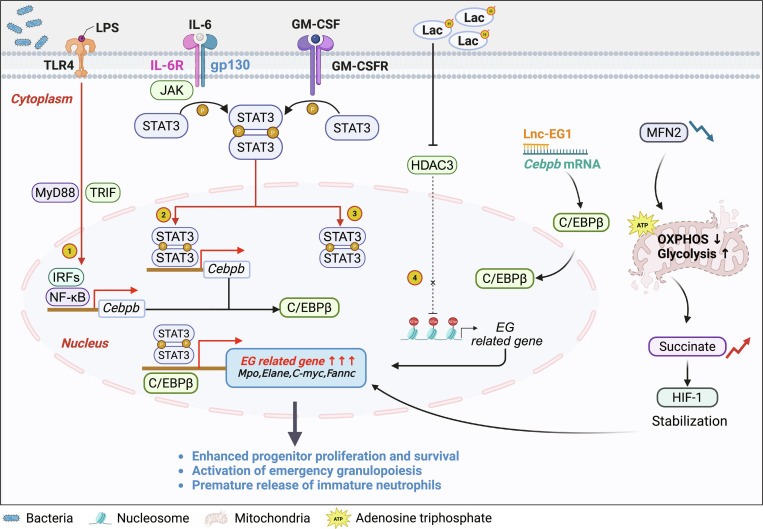
Signaling and transcriptional circuitry controlling sepsis-induced emergency granulopoiesis. Schematic of upstream inflammatory inputs and core intracellular pathways that drive emergency granulopoiesis. Systemic cues (e.g., G-CSF, IL-6, and TLR ligands) activate JAK/STAT and NF-κB in HSPCs and neutrophil progenitors, promoting lineage commitment and accelerating proliferation. The network highlights the C/EBP family, STAT3-dependent signals, and chemokine receptor rewiring that together shorten maturation, alter granule/effector programming, and enable premature egress. C/EBPε/β, CCAAT/enhancer-binding protein ε/β; STAT3, signal transducer and activator of transcription 3; TLR, Toll-like receptor; JAK, Janus kinase; NF-κB, nuclear factor κB; OXPHOS, oxidative phosphorylation. Figure created with BioRender.com.

The metabolic reprogramming of HSPCs provides an energy basis for emergency granulopoiesis by promoting myeloid gene expression. Within 24 h of sepsis, bone marrow lactate accumulation reduces histone deacetylase HDAC3 activity, tripling H3K27ac enrichment at the promoters of myeloid genes critical for granulopoiesis [e.g., myeloperoxidase (MPO) and elastase (ELANE)]. This epigenetic activation directly drives MPO/ELANE expression, fueling granulocyte differentiation. Concurrent MFN2 down-regulation fragments mitochondrial networks, further reducing oxidative phosphorylation and reinforcing glycolysis. This metabolic shift sustains adenosine triphosphate (ATP) production via hypoxia-inducible factor-1α (HIF-1α) stabilization (driven by succinate accumulation) while prioritizing myeloid lineage specification—ultimately promoting emergency granulopoiesis [18,33,39,59] (Fig. 6 and Table 2).

## Correlation between Emergency Granulopoiesis and Sepsis Prognosis

Emerging evidence underscores the critical role of immature granulocytes as dynamic clinical biomarkers that predict divergent outcomes in sepsis. Mare et al. [86] demonstrated that elevated immature granulocytes, including myelocytes, metamyelocytes, and band cells, independently predicted 28-d mortality. Drifte et al. [56] demonstrated that elevated I/T ratio is correlated with higher sequential organ failure assessment (SOFA) scores, prolonged ICU stays, and elevated mortality, surpassing traditional biomarkers such as C-reactive protein (CRP) and procalcitonin (PCT). Davenport et al. [87] categorized sepsis into SRS1 and SRS2 subsets, with SRS1 characterized by hyperactive granulopoiesis and worse outcomes. Building on this clinical stratification in humans, Kwok et al. [19] demonstrated that SRS1 endotype is marked by selective expansion of immature states, particularly immature immunosuppressive IL1R2^+^ blood subtype and cycling progenitors across multiple infectious contexts [e.g., acute respiratory distress syndrome (ARDS) and viral/bacterial sepsis].

Additionally, Liu et al. [52] employed high-dimensional flow cytometry to identify immature neutrophils in nonsurvivors, correlating these cells with severe organ failure. These cells correlate with severe organ failure and exhibit overexpression of immunosuppressive genes (PD-L1/CD274) and myeloid-derived molecules (S100A8/S100A9), implicating their roles in T cell suppression and immune paralysis. Barouni and Ostuni [28] delineated immature neutrophils into immunosuppressive (Neu1), pro-inflammatory (Neu2), and transitional (Neu3) subsets. The Neu1 subset, enriched in fatal cases, overexpressed OLFM4 and PD-L1 and was associated with organ failure. These findings imply that the “depth” of granulopoiesis, characterized by the release of early myeloid progenitors, reflects bone marrow stress and clinical deterioration (Table 3).

**Table. 3.** Relationship between emergency granulopoiesis and organ function and prognosis

Subcategory	Intervention/study index	Key findings	Refs.
Clinical cohort studies	Immature granulocytes (myelocytes, metamyelocytes, band cells)	Elevated immature granulocyte levels independently predict 28-d mortality	[86]
Clinical cohort studies	Immature-to-total neutrophil ratio (I/T ratio)	Higher I/T ratio correlates with increased SOFA scores, prolonged ICU stay, and mortality, outperforming traditional biomarkers like CRP/PCT	[56]
Sepsis subtype classification	Sepsis subtype classification (SRS1/SRS2)	SRS1 subtype (hyperactive granulopoiesis) predicts mortality across different cohorts	[86]
Single-cell subset analysis	Single-cell RNA sequencing and flow cytometry	Identification of CD10^−^CD16^low^ (immunosuppressive), S100A8/A9^high^ (pro-inflammatory) subsets; Neu1 subset (PD-L1^high^OLFM4^high^) enriched in fatal cases, associated with organ failure and T cell suppression	[42,73]
Gene knockout models	Myeloid-specific STAT3 knockout mice	Impaired progenitor cell proliferation, reduced immature granulocytes, alleviated liver and kidney injury	[14]
Drug intervention models	Anti-G-CSF neutralizing antibody treatment	Reduced release of immature neutrophils, alleviated lung injury	[42]
Cell transplantation models	Adoptive transfer of immature cells (CD11b^+^Ly6G^+^CD101^−^ cells)	Induce T cell anergy and multi-organ failure	[17]
Pharmacological inhibition models	Mac-1 inhibitor treatment	Immature neutrophils accumulate in pulmonary microvasculature through Mac-1-dependent retention, inducing microcirculatory compromise and hypoxemia	[43]

## Therapeutic Strategies to Restore Hematopoietic Equilibrium

Emerging therapeutic strategies for sepsis-induced emergency granulopoiesis are shifting from broad immunosuppression to precision interventions targeting specific dysregulations in the emergency granulopoiesis process. These interventions can be conceptually structured around 3 core targets: preventing premature neutrophil egress, modulating neutrophil differentiation and function, and reprogramming neutrophil metabolism. However, each strategy must be carefully evaluated for potential immunosuppressive trade-offs, particularly when targeting key cytokines like GM-CSF.

### Targeting neutrophil egress

Preventing the premature release of immature neutrophils from the bone marrow is a key therapeutic strategy. CXCR2 antagonists, such as reparixin, can effectively block the egress of immature neutrophils, retaining them in the marrow for further maturation. This approach has shown promise in preclinical models, associated with a reduction in peripheral immature neutrophil counts and alleviated lung injury [88–90]. A critical consideration for this approach is the timing of intervention, as delayed neutrophil recruitment might compromise initial bacterial clearance, underscoring the need for precise timing in therapeutic application.

### Modulating neutrophil differentiation and function

Correcting the pathological differentiation and function of neutrophils is a promising approach. Targeting the GM-CSF receptor (GM-CSFR) has shown potential in modulating myeloid differentiation, as interventions with GM-CSFR-targeting agents, such as tofacitinib, can reduce the pathological skewing of myeloid cells. By inhibiting GM-CSFR signaling, these agents can dampen the differentiation and expansion of granulocytes, which is particularly beneficial in conditions characterized by excessive neutrophil production [33] . However, the inhibition of GM-CSF signaling must be approached with caution. While it can dampen pathological inflammation, broad inhibition risks compromising essential host defense, as GM-CSF is crucial for neutrophil antimicrobial functions. In contrast, ex vivo differentiation of HSPCs with GM-CSF can promote the generation of neutrophils [91], offering a potential source of adoptive therapy. Furthermore, miR-223, a key regulator of granulocyte differentiation has been shown to reduce pathogenic NETosis and cytokine production [92].

### Reprogramming neutrophil metabolism

Given the critical role of metabolic rewiring in emergency granulopoiesis, targeting these pathways presents a viable strategy. Metabolic modulators that target key enzymes such as PKM2 or lactate dehydrogenase A (LDHA) could potentially recalibrate hematopoietic output [93,94]. For instance, metformin, an adenosine monophosphate kinase (AMPK) activator, can suppress glycolysis and modulate neutrophil hyperinflammatory responses [19,89,95,96]. Additionally, enhancing mitochondrial antioxidant defense, such as overexpression of SOD in induced pluripotent stem cell-derived HSPCs, has shown promise in improving neutrophil function and overall immune reconstitution [59] .

### Critical considerations and future challenges

Translating these strategies into viable therapies is fraught with significant challenges, paramount among them being the double-edged nature of immunomodulation in the dynamic context of sepsis. For example, although broadly inhibiting GM-CSF might theoretically suppress the pathological inflammation, it can impair neutrophil functions essential for antimicrobial defense, such as phagocytosis and ROS production. This could predispose patients to secondary infections and worsen outcomes during the late immunosuppressive phase of sepsis [97]. As demonstrated in clinical trials, GM-CSF administration has been shown to reverse monocytic immunoparalysis (measured by mHLA-DR expression), improving outcomes in certain patient subgroups. This highlights that therapy beneficial for one endotype could be harmful for another. Future efforts should prioritize personalized medicine strategies that selectively suppress maladaptive granulopoiesis while preserving protective immunity. The success of these interventions will depend on our ability to tailor treatments based on patient-specific factors and biomarkers, ensuring optimal outcomes while minimizing immunosuppressive risks [14,47,97,98] (Table 4).

**Table. 4.** Therapeutic strategies to restore hematopoietic equilibrium

Subcategory	Objective	Interventions	Key evidence	Refs.
Targeting neutrophil egress	Block CXCR2-mediated premature marrow egress	CXCR2 antagonists (e.g., reparixin; CXCR1/2 blockade)	↓ Circulating immature neutrophils; mitigated lung injury in models; early human safety/anti-inflammatory signals	85–87
Modulating differentiation/function	Correct myeloid skewing; generate competent neutrophils; curb NETosis	GM-CSFR modulation; GM-CSF-based ex vivo HSPC→neutrophil; miR-223	Attenuates excessive granulopoiesis; yields mature neutrophils ex vivo; reduces pathogenic NETs without major loss of killing	30, 88-89
Reprogramming metabolism	Reverse glycolytic bias and mitochondrial dysfunction	PKM2/LDHA inhibition; metformin (AMPK); mitochondrial antioxidants (e.g., SOD2)	Recalibrates hematopoietic output; dampens hyper-inflammation; improves neutrophil fitness in models	54, 86, 90-93
Critical considerations and future challenges	Stage/endotype-specific balance of emergency granulopoiesis suppression versus host defense	Biomarker-guided, time-limited modulation; GM-CSF for immunoparalysis	Broad GM-CSF inhibition risks phagocytic/ROS deficits; GM-CSF restores mHLA-DR in selected subgroups	94-95

To preserve host defense while mitigating emergency granulopoiesis-linked pathology, we advocate a selective, state-centric approach guided by time window, phenotype + function, and anatomical site. Early, short-course measures to curb premature egress and hyper-recruitment (e.g., time-limited CXCR2 antagonism) are favored [88], whereas late or broad suppression—especially blanket GM-CSF blockade—is discouraged given the risk of immunoparalysis [91–93]. Targeting is restricted to immature/pathological states (e.g., CD10^−^CD16^low^ with enrichment of PD-L1, IL1R2, and ARG1 [52,53]), only when functional immunosuppression, excess NETosis, or coagulopathic tissue injury is documented [65].

## Concluding Remarks and Future Perspectives

Sepsis-induced emergency granulopoiesis is a critical compensatory mechanism that enhances host defense by accelerating neutrophil production via bone marrow myelopoiesis. Nonetheless, this adaptive response is characterized by a significant duality: While its regulated activation mitigates pathogen spread, its dysregulated activation leads to the pathological release of immature neutrophils [18,51]. These functionally heterogeneous populations drive the pathogenesis of multiorgan injury and immunosuppression [98]. Consequently, the modulation of granulopoiesis emerges as a crucial therapeutic target in sepsis. Despite recent advancements in elucidating molecular mechanisms and clinical correlations, the urgent priorities remain to decode the complex regulatory networks and translate these insights into effective therapies [18,26,27,88].

### Limitations and challenges in current research

Significant gaps persist in understanding the initiation and termination signals regulating emergency granulopoiesis [18]. Key limitations include incomplete characterization of pathogen-specific pattern recognition pathways (e.g., differential activation of TLR/RIG-I by Gram-positive bacteria, Gram-negative bacteria, and viruses) and their downstream effects on hematopoietic reprogramming [27,59,99]. Additionally, the synergistic roles of components within bone marrow niche components, such as stromal cells, neural signaling, and metabolic regulators, require comprehensive investigation [24]. Moreover, the roles of epigenetic modifiers, such as DNA methylation, histone deacetylation, and noncoding RNA networks, in directing hematopoietic progenitor differentiation remain poorly defined [24,51]. The heterogeneity of immature neutrophils adds an additional layer of complexity and presents further challenges for mechanistic elucidation. While single-cell technologies have enabled identification of distinct subsets, their developmental trajectories, tissue-specific migratory patterns, and interactions with adaptive immune cells still remain unclear [14,19,30,31,33,47]. Understanding functional state transitions, such as hyperinflammatory-to-immunosuppressive shifts and microenvironmental adaptation, is essential to unravel sepsis pathogenesis [20].

### Future directions and translational potential

Emergency granulopoiesis in sepsis offers both diagnostic and therapeutic opportunities. Although the I/T ratio demonstrates prognostic potential, multicenter validation of standardized quantification protocols is needed. Therapeutic strategies should balance antimicrobial defense with tissue protection through phase-specific interventions tailored to individual immune states. Four strategies might address these challenges: (a) High-resolution cellular tracking: Combine in vivo single-cell fate mapping with real-time intravital imaging to resolve spatiotemporal trajectories of neutrophil development, egress, and tissue entry in pathological neutrophil. (b) Translational models: Engineer humanized murine systems and ex vivo bone marrow organoids that recapitulate sepsis-induced hematopoietic heterogeneity and allow reversible, mechanism-specific perturbations aligned to state-centric hypotheses. (c) Multi-omics data integration: Integrate transcriptomic, epigenomic, proteomic, and metabolomic layers with standardized functional readouts to infer regulatory hierarchies and nominate function-sparing nodes for intervention. (d) Precision clinical trials: Design biomarker-stratified, short-course, site-directed trials that enroll patients with concordant phenotype + function criteria, and test selective modulators under predefined stopping rules alongside antimicrobial/source control.

In conclusion, research on sepsis-related granulopoiesis is evolving from descriptive studies to mechanism-based therapeutic approaches. Integrating fundamental discoveries with clinical applications across a comprehensive understanding of the hematopoietic–immune–organ axis may help preserve host defense while mitigating emergency granulopoiesis-linked pathology.
